# A nomogram for predicting cancer-specific survival in patients with uterine clear cell carcinoma: a population-based study

**DOI:** 10.1038/s41598-023-36323-w

**Published:** 2023-06-07

**Authors:** Wen-li Cheng, Rui-min Wang, Yi Zhao, Juan Chen

**Affiliations:** 1 Department of Outpatient, West China Second University Hospital, Sichuan University, Chengdu, China; 2 Key Laboratory of Birth Defects and Related Diseases of Women and Children (Sichuan University), Ministry of Education, West China Second University Hospital, Sichuan University, Chengdu, Sichuan China

**Keywords:** Cancer therapy, Endometrial cancer

## Abstract

Uterine clear cell carcinoma (UCCC) is a relatively rare endometrial cancer. There is limited information on its prognosis. This study aimed to develop a predictive model predicting the cancer-specific survival (CSS) of UCCC patients based on data from the Surveillance, Epidemiology, and End Results (SEER) database between 2000 and 2018. A total of 2329 patients initially diagnosed with UCCC were included in this study. Patients were randomized into training and validation cohorts (7:3). Multivariate Cox regression analysis identified that age, tumor size, SEER stage, surgery, number of lymph nodes detected, lymph node metastasis, radiotherapy and chemotherapy were independent prognostic factors for CSS. Based on these factors, a nomogram for predicting the prognosis of UCCC patients was constructed. The nomogram was validated using concordance index (C-index), calibration curves, and decision curve analyses (DCA). The C-index of the nomograms in the training and validation sets are 0.778 and 0.765, respectively. Calibration curves showed good consistency of CSS between actual observations and nomogram predictions, and DCA showed that the nomogram has great clinical utility. In conclusion, a prognostic nomogram was firstly established for predicting the CSS of UCCC patients, which can help clinicians make personalized prognostic predictions and provide accurate treatment recommendations.

## Introduction

Uterine clear cell carcinoma (UCCC) is a relatively rare endometrial cancer^1,2^. It was first reported by Kay in 1957^3^, and its incidence accounted for 1–5% of endometrial cancer^4,5^. Compared with endometrioid adenocarcinoma, UCCC patients are often associated with high risk factors such as advanced clinical stage, deep myometrial invasion, lymphovascular involvement, and distant metastasis, with higher recurrence and mortality^6–8^. Occult metastases occur in approximately 40–50% of UCCC initially thought to be confined to the uterus^9^. The 5-year survival rate of patients with stage II and above of the International Federation of Gynecology and Obstetrics (FIGO) has been hovering below 50% for a long time^6,8^, which is much lower than that of endometrioid adenocarcinoma^10^.

Due to the rarity of UCCC, there is limited information on its biology and pathogenesis^11–13^. There is still a lack of in-depth understanding of the treatment and prognosis of UCCC. Currently, a comprehensive treatment approach similar to the more common histological subtypes of endometrioid endometrial cancer and adenocarcinoma of the cervix is adopted for UCCC patients, including surgery with adjuvant chemoradiotherapy^9,14,15^. The FIGO and American Joint Committee on Cancer (AJCC) tumor node metastasis (TNM) staging systems are commonly used for prognostic estimates and clinical treatment in patients with UCCC. However, both staging systems had several limitations, including low accuracy, ignorance of other factors such as age, and poor performance in predicting individual survival risk^16–18^. Therefore, a personalized predictive model is needed for UCCC patients.

Accurately predicting the survival probability of an individual tumor patient may change the pattern of medical practice and aid in clinical decision-making. As a risk and benefit assessment tool that can provide physicians and patients with more objective and accurate information, clinical prediction models have been increasingly used in recent years. The nomogram is a statistical-principles-based predictive tool that integrates key predictors and is widely used to quantify risk and assess prognosis in multiple cancers^19–21^. However, to our knowledge, no nomogram has been developed for the prognosis of UCCC patients. The purpose of this study was to construct a nomogram using UCCC patient data extracted from the Surveillance, Epidemiology, and End Results (SEER) database and then validate the predictive model to determine its performance.

## Results

### Patient characteristics

**Figure 1:**
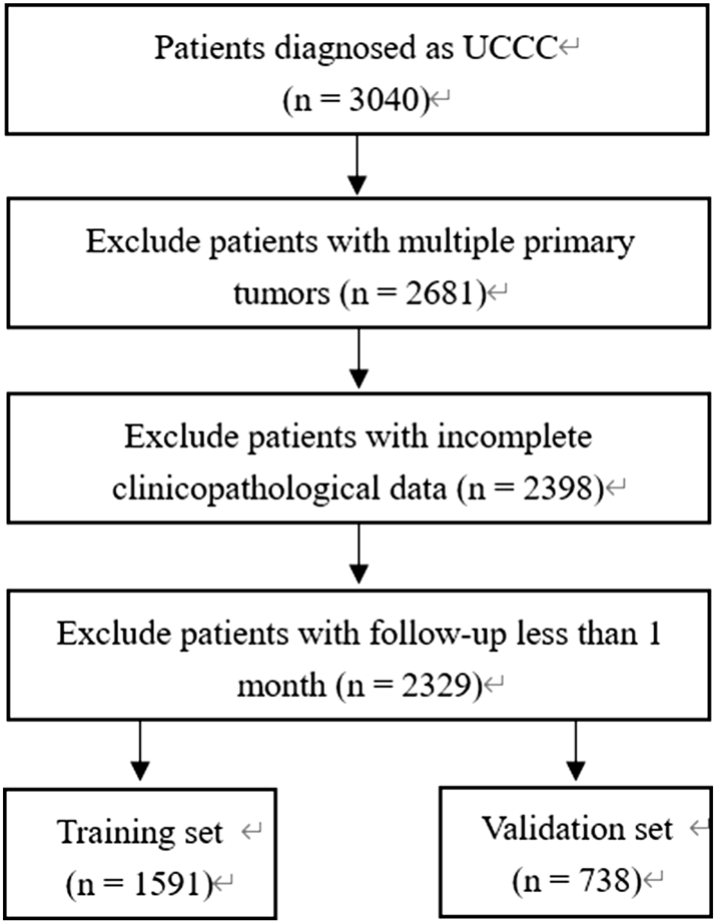
Flow chart of the data selection process.

A total of 2329 patients were finally included and randomly divided into a training cohort of 1591 and a validation cohort of 738. The data selection flow chart is shown in Fig. 1. For continuous variables, the optimal cut-off value was determined by X-Tile software, which was converted to categorical variables. Among them, the optimal cut-off values for age were 60 and 70 years, respectively, the optimal cut-off values for tumor diameter were 30 and 70 mm, and the number of detected lymph nodes was 2 and 9, respectively. The clinicopathological characteristics of the training cohort and the validation cohort are shown in Table 1, and there was no significant difference between the two groups (*p* > 0.05).

**Table 1:** Clinicopathological characteristics and treatment strategy of UCCC patients.

	Training cohort (N = 1591)	Validation cohort (N = 738)	*P*-value
Age (years)			
< 60	430 (27.0%)	221 (29.9%)	0.343
60–70	572 (36.0%)	256 (34.7%)	
> 70	589 (37.0%)	261 (35.4%)	
Race			
White	1146 (72.0%)	554 (75.1%)	0.25
Black	268 (16.8%)	116 (15.7%)	
Other	177 (11.1%)	68 (9.2%)	
Marital status			
Married	721 (45.3%)	335 (45.4%)	1
Unmarried	870 (54.7%)	403 (54.6%)	
Year of diagnosis			
2000–2008	580 (36.5%)	278 (37.7%)	0.604
2009–2018	1011 (63.5%)	460 (62.3%)	
Tumor size (mm)			
< 30	330 (20.7%)	159 (21.5%)	0.749
30–70	556 (34.9%)	243 (32.9%)	
> 70	186 (11.7%)	94 (12.7%)	
Grade			
I	23 (1.4%)	14 (1.9%)	0.184
II	89 (5.6%)	43 (5.8%)	
III	661 (41.5%)	331 (44.9%)	
IV	286 (18.0%)	105 (14.2%)	
Unknown	532 (33.4%)	245 (33.2%)	
SEER staging			
Localized	682 (42.9%)	331 (44.9%)	0.604
Regional	599 (37.6%)	263 (35.6%)	
Distant	310 (19.5%)	144 (19.5%)	
AJCC staging			
I	894 (56.2%)	424 (57.5%)	0.727
II	186 (11.7%)	85 (11.5%)	
III	390 (24.5%)	167 (22.6%)	
IV	121 (7.6%)	62 (8.4%)	
Surgery			
No	227 (14.3%)	114 (15.4%)	0.493
Yes	1364 (85.7%)	624 (84.6%)	
LNE			
< 2	514 (32.3%)	263 (35.6%)	0.131
2–9	326 (20.5%)	159 (21.5%)	
> 9	751 (47.2%)	316 (42.8%)	
LNP			
No	813 (51.1%)	364 (49.3%)	0.185
Yes	301 (18.9%)	126 (17.1%)	
No examined	477 (30.0%)	248 (33.6%)	
Radiation			
No	861 (54.1%)	394 (53.4%)	0.777
Yes	730 (45.9%)	344 (46.6%)	
Chemotherapy			
No	811 (51.0%)	384 (52.0%)	0.667
Yes	780 (49.0%)	354 (48.0%)	

*UCCC* uterine clear cell carcinoma, *LNE* number of lymph nodes examined, *LNP* lymph nodes positive.

Median follow-up was 56 months (range 1–227 months). During this period, 853 (36.6%) cancer-specific deaths occurred, and the cumulative 5- and 10-year CSS for the entire cohort were 58.8% and 54.8%, respectively.

### Construction of the nomogram

**Figure 2:**
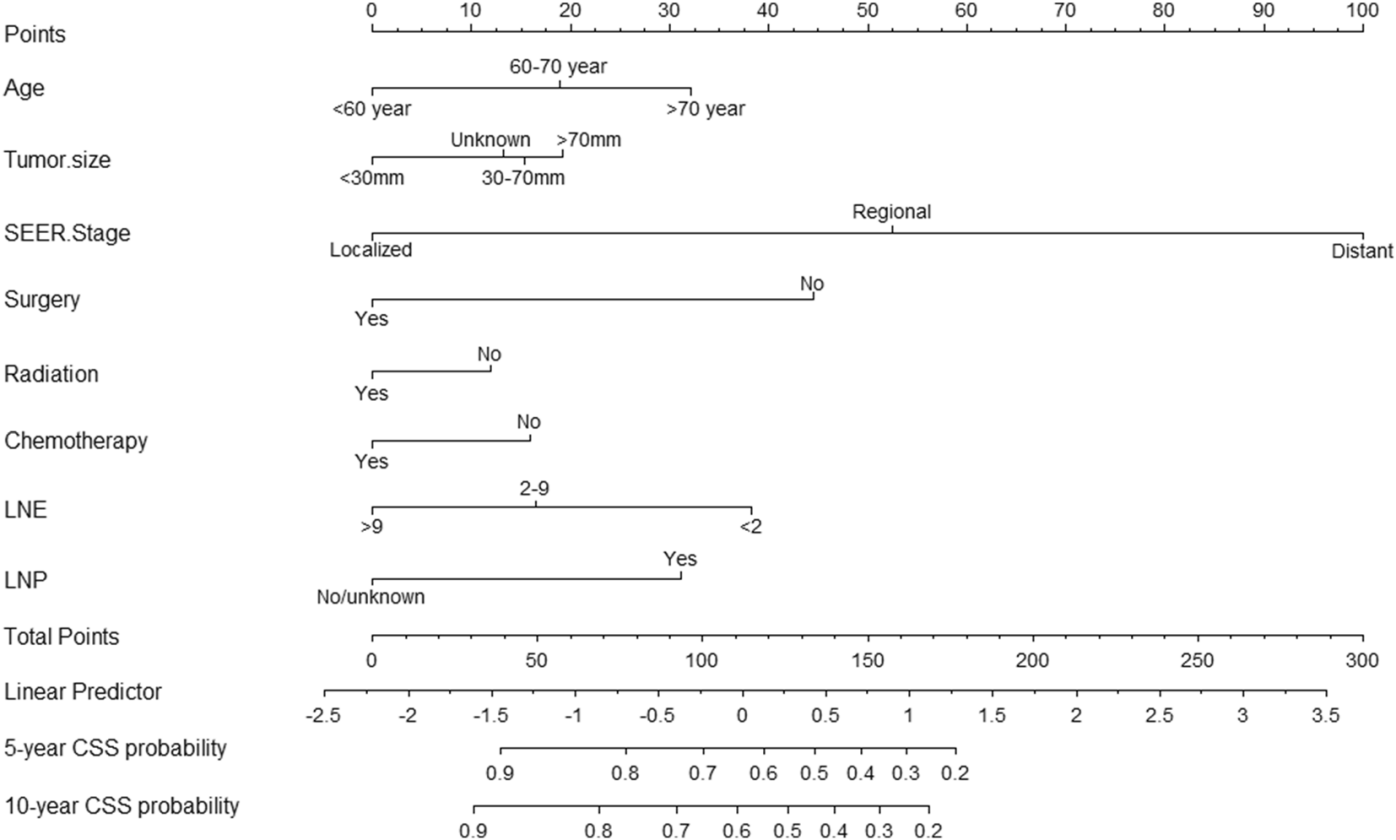
Nomogram for predicting 5- and 10-year CSS probability in patients with UCCC. *CSS* cancer-specific survival, *UCCC* Uterine clear cell carcinoma.

For the training set, the Cox univariate analysis showed that the following factors were significantly associated with CSS: age, race, marital status, tumor size, pathological grade, SEER stage, AJCC stage, surgery, number of lymph nodes detected, lymph node metastasis, radiotherapy and chemotherapy (all *p* < 0.05). The Cox multivariate regression analysis showed that age, tumor size, SEER stage, surgery, number of lymph nodes detected, lymph node metastasis, radiotherapy and chemotherapy were independent prognostic factors for CSS (Table 2). In the multi-collinearity analysis performed among these variables, all VIFs were less than 2 (data not shown). This result revealed that there was no multi-collinearity between these variables. According to the above clinicopathological factors, a personalized nomogram for predicting the prognosis of UCCC patients was successfully constructed, and SEER stage had the greatest impact on the prognosis of UCCC patients. After the clinician entered the clinicopathological information of a specific UCCC patient into the nomogram, the corresponding score on the scoring scale was obtained, and the obtained score was added to the total subscale. Finally, drawing a vertical line on the survival scale gives the patient's 5- and 10-year probability of survival (Fig. 2).

**Table 2:** Univariate and multivariate analyses of cancer-specific survival in the training cohort.

Characteristics	Univariate analysis		*P* value	Multivariate analysis		*P* value
	HR	95% CI		HR	95% CI	
Age (years)						
< 60	1.00			1.00		
60–70	1.26	1.01–1.56	0.04	1.32	1.05–1.65	0.016
> 70	1.75	1.42–2.16	< 0.001	1.72	1.37–2.16	< 0.001
Race						
White	1.00					
Black	1.47	1.20–1.8	< 0.001			
Other	0.68	0.50–0.92	0.013			
Marital status						
Married	1.00					
Unmarried	1.40	1.19–1.65	< 0.001			
Site recode						
Corpus uteri	1.00					
Cervix uteri	0.91	0.73–1.13	0.394			
Uterus, NOS	1.19	0.59–2.39	0.631			
Year of diagnosis						
2000–2008	1.00					
2009–2018	1.01	0.86–1.19	0.911			
Tumor size (mm)						
< 30	1.00			1.00		
30–80	2.31	1.73–3.09	< 0.001	1.40	1.03–1.88	0.029
> 80	4.32	3.13–5.96	< 0.001	1.58	1.12–2.23	0.008
Unknown	3.07	2.30–4.08	< 0.001	1.44	1.06–1.95	0.021
Grade						
I	1.00					
II	4.25	1.01–17.79	0.048			
III	5.79	1.43–23.46	0.014			
IV	6.46	1.61–25.95	0.009			
Unknown	6.56	1.63–26.44	0.008			
SEER stage						
Localized	1.00			1.00		
Regional	3.23	2.57–4.05	< 0.001	2.58	1.41–4.73	0.002
Distant	11.21	8.87–14.17	< 0.001	7.39	5.51–9.91	< 0.001
AJCC stage						
I	1.00					
II	0.95	0.72–1.25	0.7			
III	1.67	1.38–2.01	< 0.001			
IV	4.28	3.3–5.55	< 0.001			
Surgery						
No	1.00			1.00		
Yes	0.20	0.17–0.24	< 0.001	0.38	0.30–0.48	< 0.001
LNE						
< 2	1.00			1.00		
2–9	0.43	0.34–0.53	< 0.001	0.65	0.41–1.03	0.067
> 9	0.26	0.22–0.31	< 0.001	0.50	0.32–0.78	0.002
LNP						
No	1.00			1.00		
Yes	3.34	2.68–4.16	< 0.001	1.48	1.13–1.95	0.005
No examined	4.63	3.81–5.63	< 0.001	0.91	0.57–1.45	0.683
Radiation						
No	1.00			1.00		
Yes	0.81	0.69–0.96	0.012	0.83	0.69–0.99	0.041
Chemotherapy						
No	1.00			1.00		
Yes	1.36	1.16–1.60	< 0.001	0.69	0.57–0.84	< 0.001

*UCCC* uterine clear cell carcinoma, *LNE* number of lymph nodes examined, *LNP* lymph nodes positive.

### Validation of the nomogram

**Figure 3:**
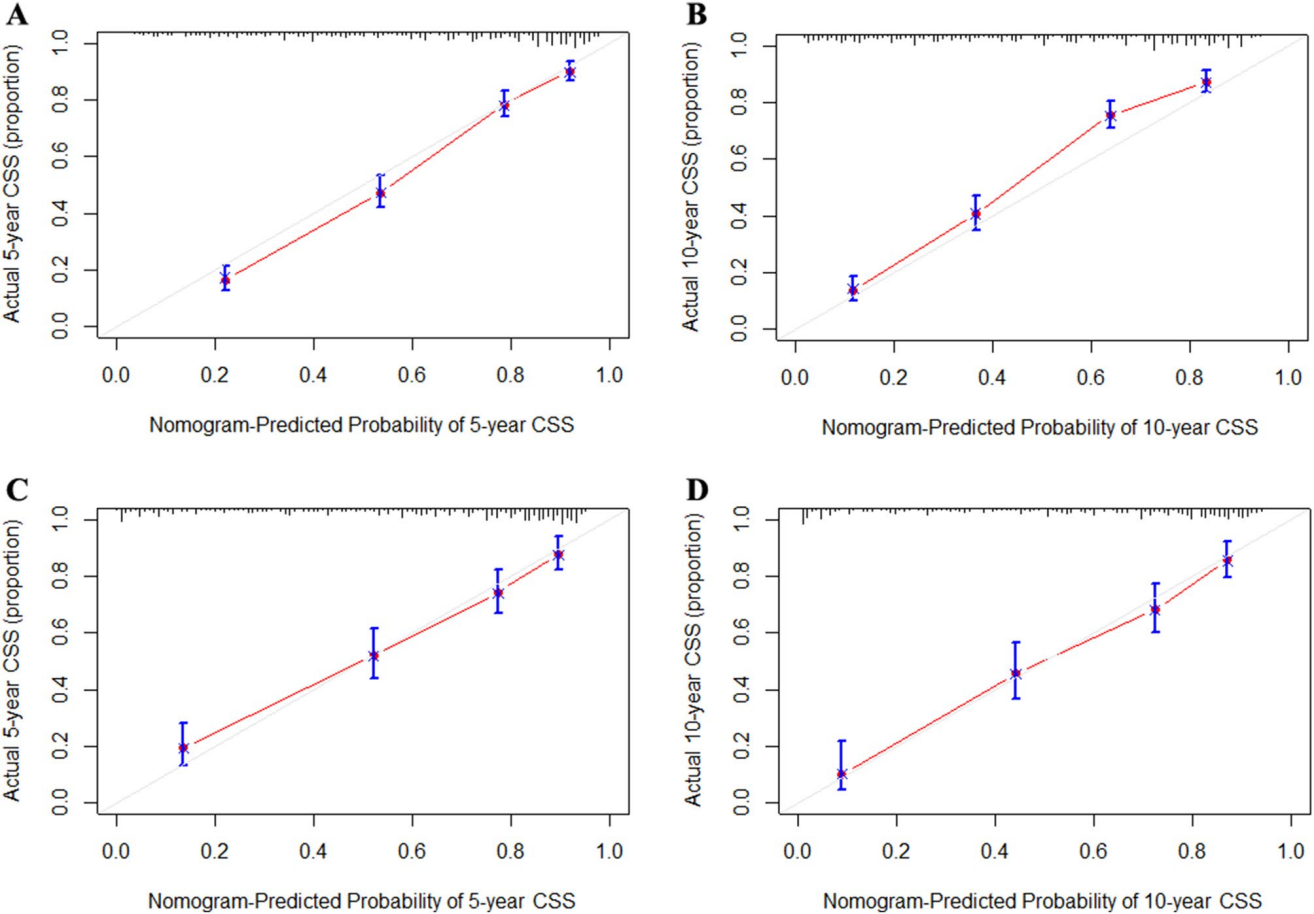
Calibration curves of the nomogram. (**A**,**B**) Calibration curves of 5-year and 10-year CSS for UCCC patients in the training cohort. (**C**,**D**) Calibration curves of 5-year and 10-year CSS for UCCC patients in the validation cohort. *CSS* cancer-specific survival, *UCCC* Uterine clear cell carcinoma.

**Figure 4:**
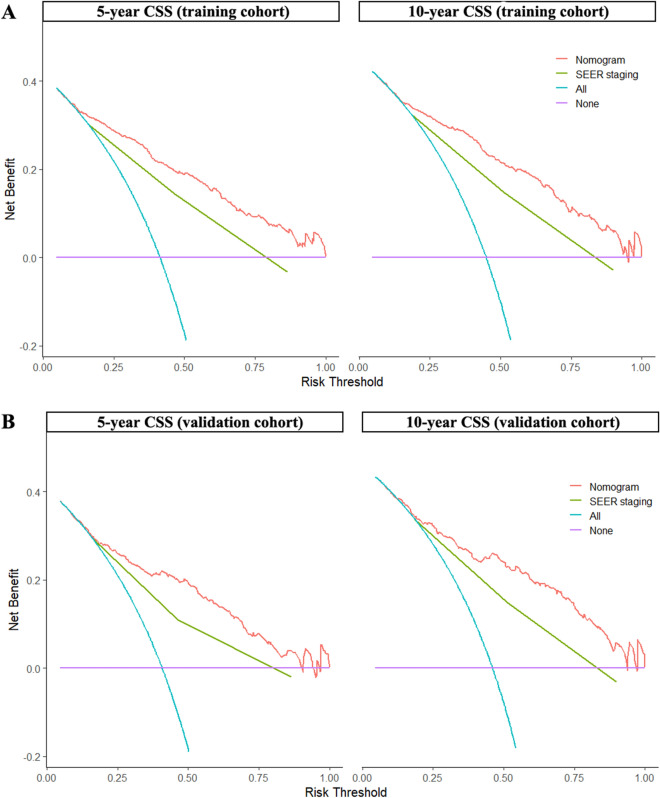
Decision curves of the nomogram. (**A**) 5-year and 10-year CSS benefit in the training cohort. (**B**) 5-year and 10-year CSS benefit in the validation cohort. *CSS* cancer-specific survival.

The C-index of the nomogram in the training set and validation set is 0.778 (95% CI 0.758–0.798) and 0.765 (95% CI 0.743–0.787), respectively, indicating that the nomogram has good prediction accuracy. Calibration curve analysis showed that the survival rate predicted by the nomogram was in good agreement with the actual survival rate, indicating that the nomogram had better predictive performance (Fig. 3). DCA showed that at nearly all threshold probabilities, using the established nomogram for predicting outcomes in UCCC patients provided a greater net benefit than the "all or zero deaths in all patients" strategy, suggesting that the nomogram has potential clinical applicability. Furthermore, DCA showed that the nomogram model curve was higher than the SEER stage curve, indicating that the nomogram model was superior to the SERR staging system (Fig. 4).

## Discussion

In this study, we developed a nomogram for predicting CSS in UCCC patients based on eight predictors of patient’s age, tumor size, SEER stage, surgery, number of lymph nodes detected, lymph node metastasis, radiotherapy and chemotherapy. The predictors included in the model can be easily obtained from clinical practice. Validation of the model using different statistical methods demonstrates its excellent performance. Furthermore, DCA demonstrated that our nomogram predicted survival with better clinical benefit and utility than the conventional staging system.

UCCC is rare and considered to be prone to myometrial invasion, lymphovascular invasion, lymph node metastasis and extrauterine metastasis, so most of them were diagnosed at a later stage. Due to its rarity, there are few studies on UCCC, and these studies are usually single-center, small-sample studies^13,22–24^, thus there is currently a lack of high-quality evidence-based evidence on its biological characteristics, optimal treatment options, and prognostic assessment. At present, in clinical practice, obstetricians and gynecologists often evaluate the prognosis of UCCC patients and formulate follow-up treatment plans according to the patient's AJCC or FIGO stage, pathological grading, and intraoperative conditions^9,14,15^. However, this method mostly relies on the clinical experience of physicians, and cannot conduct a more comprehensive survival analysis and prognosis evaluation according to the patient's disease characteristics. Therefore, a more systematic diagnosis and treatment plan and prognostic risk assessment for UCCC are urgently needed.

Previous studies indicated that age, tumor size and pathologic stage might be important factors affecting the prognosis of UCCC^22–24^. However, due to the small number of cases in these studies, the conclusions are inconsistent. In this study, based on national data from a relatively large cohort, our study found that age, tumor size, SEER stage, surgery, number of lymph nodes detected, lymph node metastasis, radiotherapy and chemotherapy were significantly correlated with the prognosis of UCCC. Among them, SEER stage is the most important factor affecting the prognosis of patients. The higher the SEER stage, the worse the prognosis of the patient. Surgery is the second important factor on the survival rate of UCCC patients based on the nomogram. Currently, total hysterectomy plus bilateral adnexectomy plus pelvic and para-aortic lymph node dissection have been established as first-line treatment^23,25^. This comprehensive staging surgery can better perform accurate staging and provide a reference for subsequent selection of appropriate adjuvant therapy. Lymph node metastasis is one of the main factors affecting the prognosis of patients with endometrial cancer. However, the effect of lymphadenectomy on the survival of UCCC patients remains controversial. In many studies, systematic lymph node dissection has resulted in better outcomes for patients with UCCC^14,26^. Conversely, other studies have shown that lymphadenectomy has no prognostic value^2,22^. One reason for this discrepancy may be that the number of lymph nodes dissected was not taken into account. Our study observed that patients with more than 9 lymph nodes removed had better CSS than those with < 2 lymph nodes removed. However, due to the lack of information on the extent of lymph node dissection, we can't compare the effects of systematic lymphadenectomy with less extensive lymphadenectomy (such as sentinel lymph node dissection or sampling) on the prognosis, which needs further improvement in future research. Age is an independent prognostic factor for UCCC, which is consistent with previous studies^23,27^. In addition, multivariate analysis showed that radiotherapy and chemotherapy were also protective factors affecting the prognosis of UCCC patients.

This is the first study to established a prognostic model for UCCC. Based on the SEER database system, this study integrated the relevant clinicopathological factors and treatment patterns affecting the prognosis of UCCC patients into a nomogram, thereby successfully constructing a predictive model consistent with the condition of UCCC patients. Compared with the SEER staging system (surrogate for traditional FIGO staging), it has the advantages of being comprehensive, intuitive, more accurate and convenient. The multi-center large sample also provides a guarantee for the credibility of the final model.

This study has several limitations. First, SEER database lacks detailed information about chemotherapy and radiotherapy, and there is no data about surgical margins, extent of pelvic lymph node dissection, and lymph node invasion, which may affect the prognosis of UCCC. Second, the nomogram model is only verified internally. It is necessary to use cohort and prospective randomized clinical trials from other countries for external verification to confirm its performance. Third, there may be selection bias due to the nature of retrospective analysis.

In conclusion, we developed a nomogram for predicting CSS in UCCC patients based on the SEER database, which can help clinicians make individualized prognosis predictions and provide accurate treatment recommendations.

## Methods

### Patient selection

Data on UCCC patients registered between 2000 and 2018 were extracted from the SEER database using SEER* Stat (version 8.4.0.1) software. Inclusion criteria included: (1) pathologically confirmed UCCC, coded as 8310/3 according to the International Classification of Diseases for Oncology, Third Edition (ICD-O-3); (2) primary site included corpus uteri/uterus not specified; (3) age ≥ 18 years old. Exclusion criteria included: (1) multiple primary tumors; (2) incomplete clinicopathological data; (3) lost to follow-up or follow-up less than 1 month.

Extracted data included: gender, age, race, marital status, tumor location, tumor size, year of diagnosis, pathological grade, SEER stage, AJCC TNM staging (7th edition), surgery, chemoradiotherapy, follow-up time and survival. The SEER stage (local, regional, and distant) was used to classify the extent of the disease as a surrogate for the traditional FIGO staging. The primary endpoint of the study was cancer-specific survival (CSS), defined as the time from diagnosis to death from UCCC or time to last follow-up. The optimal cutoff values for continuous variables were determined using the "X-Tile" software (Yale School of Medicine, CT, USA), converting age, tumor size, number of lymph nodes dissected into categorical variables.

### Statistical analysis

The final included UCCC patients were randomly assigned to the training set and the validation set in a 7:3 ratio using R software. The training set was used to build a risk prediction model and to construct a nomogram to predict a patient's CSS at 5 and 10 years. Validation groups are used for internal validation. For comparison of count data between groups, chi-square or Fisher's exact test is used; for comparison of multi-category variables between groups, chi-square test or Fisher's exact probability method for R*C tables is used. Continuous variables were compared using the t-test or the Mann–Whitney U test. In the training group, univariate and multivariate analyses were performed by Cox proportional hazards regression models to identify independent prognostic factors associated with CSS. The patient characteristics with *p* < 0.05 in univariate analysis were included in multivariate analysis. A nomogram model was constructed based on the independent prognostic factors defined in the multivariate analysis. Meanwhile, the variance inflation factor (VIF) was assessed among the covariates in the nomogram, and VIF > 4.0 was interpreted as indicating multicollinearity. Variables with VIF greater than 4.0 were not included in the final model analysis. The discrimination and consistency of the model were evaluated by the consistency index (C index) and the calibration curve (1000 cycles by the bootstrap method). The larger the C index, the more accurate the prognosis prediction. Calibration curves are used to describe the difference between predicted probabilities and actual outcomes. The x-axis represents predicted survival time and the y-axis represents actual survival time. In a perfect forecasting model, the forecast rate would decline along a 45° slope. The clinical utility of nomograms was assessed by applying decision curve analysis (DCA) to calculate the net gain over a range of threshold probabilities. The y-axis represents net gain and the x-axis represents threshold. All statistical analyses were performed using R software version 4.1.3. *P* < 0.05 means the difference is statistically significant.

### Ethics approval and consent to participate

Approval was waived by the local ethics committee, as SEER data is publicly available and de-identified.

## Data Availability

The data that support the findings of this study are openly available in software package SEER*Stat 8.4.0.1 (https://seer.cancer.gov/seerstat/).
